# The association of unplanned pregnancy with perinatal depression: a longitudinal cohort study

**DOI:** 10.1007/s00737-022-01225-9

**Published:** 2022-03-26

**Authors:** Lotte Muskens, Myrthe G. B. M. Boekhorst, Willem J. Kop, Marion I. van den Heuvel, Victor J. M. Pop, Annemerle Beerthuizen

**Affiliations:** 1 Department of Medical and Clinical Psychology, Tilburg University, Warandelaan 2, 5037 AB Tilburg, Netherlands; 2 Department of Cognitive Neuropsychology, Tilburg University, Tilburg, Netherlands; 3 Department of Psychiatry, section Medical Psychology and Psychotherapy, Erasmus MC, Rotterdam, Netherlands

**Keywords:** Unplanned pregnancy, Depression, Edinburgh Postnatal Depression Scale, Perinatal, Longitudinal, Mixed models

## Abstract

Perinatal depression is common, affecting approximately 7–13% of women. Studies have shown an association between unplanned pregnancy and perinatal depressive symptoms, but many used a cross-sectional design and limited postnatal follow-up. The current study investigated the association of unplanned pregnancy with perinatal depressive symptoms using a longitudinal cohort study that followed women from the first trimester until 12 months postpartum. Pregnant women (*N* = 1928) provided demographic and clinical data and information about pregnancy intention at the first trimester. Depressive symptoms were assessed during each trimester of pregnancy and five times postpartum using the Edinburgh Postnatal Depression Scale (EPDS) until 12 months postpartum. Mixed model analyses were used to investigate the association between an unplanned pregnancy and the level of depressive symptoms. Women with an unplanned pregnancy (*N* = 111, 5.8%) reported persistently higher levels of depressive symptoms during the entire perinatal period compared to women with a planned pregnancy, after adjustment for confounders (*p* < 0.001). However, the *course* of depressive symptom scores over time in women with an unplanned pregnancy was similar to that of women with a planned pregnancy. Lower age (*p* = 0.006), unemployment (*p* = 0.004), and history of depression (*p* < 0.001) were significantly associated with higher levels of perinatal depressive symptoms. An unplanned pregnancy may have a long-lasting negative impact on a woman’s perinatal mental health. Therefore, women with an unplanned pregnancy may benefit from systematic follow-up during the perinatal period with contingent mental health support.

## Introduction

Perinatal depression is a common problem affecting approximately 7–13% of women (Bennett et al. 2004; Gavin et al. 2005; Woody et al. 2017). In the current study, the perinatal period was defined as the period during pregnancy up to 12 months postpartum. Perinatal depressive symptoms have not only been linked to severe obstetric complications such as pregnancy-induced hypertension, preterm birth, and low birth weight (Grote et al. 2010; Shay et al. 2020; Staneva et al. 2015), but also to impaired neurodevelopment, lower cognitive development, and later psychopathology in the offspring (Liu et al. 2017; Van den Bergh et al. 2017). Factors associated with an increased risk of depressive symptoms during the perinatal period include, among others, lower level of education, lower income, smoking, previous history of mental health problems, having no partner, and pregnancy loss (Beck 2001; Biaggi et al. 2016; Howard et al. 2014; Lancaster et al. 2010; Robertson et al. 2004).

Unplanned pregnancy is another important predictor of perinatal depression (e.g., (Beck 2001; Biaggi et al. 2016; Lancaster et al. 2010). The terms unplanned, unintended, mistimed, and unwanted pregnancy are often used interchangeably in the literature, even though there is an important difference between pregnancies that occur two or more years earlier than desired (mistimed), in contrast to pregnancies that are not wanted at all (unwanted) (Goossens et al. 2016; Sedgh et al. 2014). Less planned pregnancies have been related to several factors such as multiparity, low level of education, not having a partner, experiencing intimate partner violence, and a history of drug abuse (Goossens et al. 2016). Furthermore, an unplanned pregnancy seems to negatively impact adequate antenatal care during pregnancy. For example, according to a meta-analysis by Dibaba et al. (2013b), women with an unplanned pregnancy enter prenatal care later and tend to have fewer prenatal visits. These women also report poorer lifestyle habits during the perinatal period, such as no or lower intake of folic acid before pregnancy and lower vitamin intake during pregnancy, continuation of smoking and alcohol consumption during pregnancy, lower relationship satisfaction, and lower levels of social support (Goossens et al. 2016; Hill et al. 2019). These observations suggest that women with an unplanned pregnancy might take less care of themselves and their unborn child during the perinatal period.

Furthermore, several studies have confirmed that there is an association between unplanned pregnancy and higher levels of depressive symptoms. For example, Boekhorst et al. (2019) concluded that unplanned pregnancies are associated with persistently higher levels of depressive symptoms during the course of pregnancy. Other studies have assessed this association between unplanned pregnancy and depressive symptoms both during pregnancy and postpartum. For example, a prospective Brazilian study found that women with an unplanned pregnancy were 2.5 times more likely to have a depression during pregnancy and the postpartum period (11 months postpartum), compared to women with a planned pregnancy (Faisal-Cury et al. 2017). In addition, in their systematic review and meta-analysis, Abajobir et al. (2016) reported that women with an unintended pregnancy had a two-fold higher risk of developing perinatal depressive symptoms compared to women with an intended pregnancy. Only few studies focused on the possible association between unplanned pregnancy and the occurrence of depressive symptoms throughout the entire perinatal period (during pregnancy up to 12 months postpartum). Christensen et al. (2011) showed that unintended pregnancy (defined as intended, mistimed, and unwanted pregnancy) was associated with a pattern of high levels of postpartum (6 weeks to 12 months) depressive symptoms but not a pattern of high symptom levels during the course of pregnancy. Another longitudinal study found that women with an unwanted pregnancy (defined as an unplanned *and* unwanted pregnancy and a negative first reaction to the pregnancy), reported slightly higher levels of depressive symptoms in the earlier phases of pregnancy, but this difference diminished during the follow-up period, both 3–5 days and 6 months postpartum (Najman et al. 1991). These two studies both used different instruments, the Beck Depression Inventory-II and the Delusions-Symptoms-States Inventory, to assess depressive symptoms. Furthermore, both studies did not cover the postnatal period from pregnancy until 12 months postpartum. Christensen et al. (2011) measured postnatal depressive symptoms during three follow-ups at 6 weeks, 4 months, and 12 months postpartum, and Najman et al. (1991) used two postnatal measurements, namely at 3–5 days after childbirth and 6 months postpartum.

Some of these studies on the relationship between unplanned pregnancy and perinatal depressive symptoms are limited by a cross-sectional design, in which data is collected during a single moment in time. For example, McCrory and McNally (2013) found that unintended pregnancy was associated with increased risk of depression at 9 months postpartum, and Dibaba et al. (2013a) found that an unwanted pregnancy is associated with an elevated risk of depression, measured once during pregnancy. On the other hand, with a longitudinal design (e.g., Christensen et al. 2011; Najman et al. 1991), data are collected during repeated observations of the same group, allowing to assess changes in individuals over time. Longitudinal assessments seem of great importance to gain a better understanding of the role of an unplanned pregnancy in the occurrence of perinatal depressive symptoms, since perinatal depressive symptoms tend to be variable over time (Baron et al. 2017). Moreover, a recent study showed that postnatal depression can persists long after birth — for one quarter of women even until 3 years postpartum (Putnick et al. 2020). To confirm the association between unplanned pregnancy and increased depressive symptom levels in pregnant and postpartum women, a study with sufficient epidemiological power with multiple assessments of depressive symptoms over the entire perinatal period is needed.

The current study assessed the association of unplanned pregnancy with the longitudinal course of depressive symptoms, at all trimesters of pregnancy and five times postpartum during the first postpartum year and tested whether the trajectories of depressive symptoms developed differently for women with an unplanned versus a planned pregnancy. It was hypothesized that women with an unplanned pregnancy report higher levels of depressive symptoms throughout their pregnancy and the postnatal period compared to women with a planned pregnancy.

## Materials and methods

### Participants and procedure

The current study is part of a large longitudinal cohort study, the Holistic Approach to Pregnancy and the first Postpartum Year (HAPPY) study (Truijens et al. 2014). Seventeen participating community midwife practices in the south of the Netherlands invited women to participate during their first antenatal appointment. Inclusion criteria were enrollment during the first trimester of pregnancy and a sufficient understanding of the Dutch language to complete the questionnaires. Exclusion criteria were multiple pregnancy, a history of chronic disease (e.g., diabetes, thyroid dysfunction), a severe psychiatric disorder (schizophrenia, borderline personality disorder or bipolar disorder), HIV, drug or alcohol addiction, or any other disease treated with medications that are potentially harmful for the fetus and need careful follow-up during pregnancy. In total, 3160 pregnant women were invited to participate between January 2013 and September 2014. Of these women, 2269 (72%) participated and provided written informed consent.

**Fig. 1:**
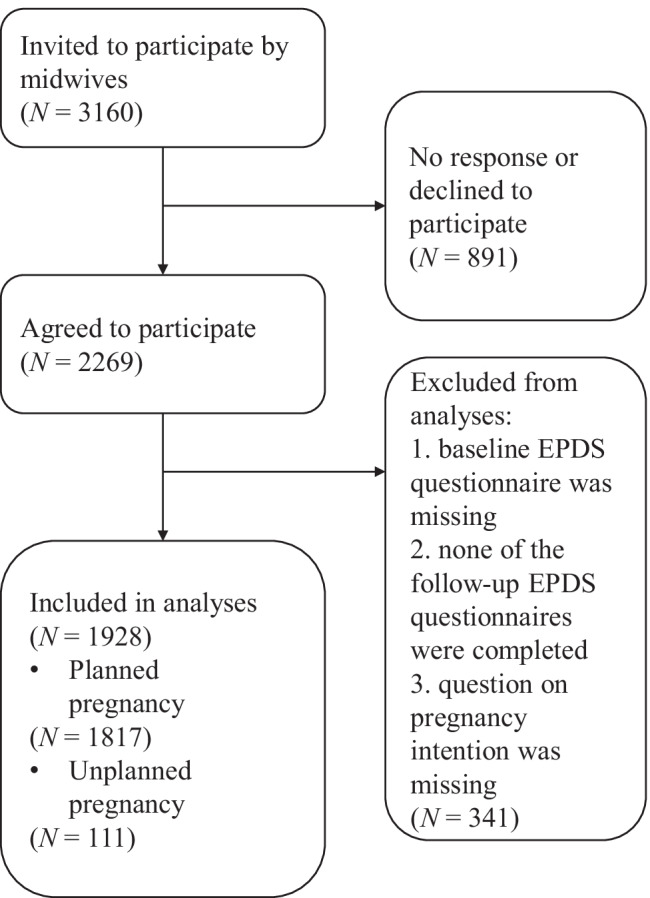
Flowchart for the participating women in the current study

In each trimester of pregnancy (12, 22, and 32 weeks of pregnancy) and postpartum (1 week, 6 weeks, 4 months, 8 months, and 12 months postpartum) participating women received (online) questionnaires. For mixed model analyses, all cases can be included, including those with missing data (Bagiella et al. 2000) (see statistical analyses for details). We only included women who (1) at least completed the Edinburgh Postnatal Depression Scale (EPDS) baseline questionnaire (12 weeks of pregnancy); (2) at least completed one of the follow-up EPDS questionnaires; and (3) answered the question regarding an (un)planned pregnancy. This resulted in a final sample of 1928 women to be included for analyses (Fig. 1).

The Ethics Review Board of Tilburg University (protocol number EC-2012.25) approved the HAPPY study. The study was reviewed by the Medical Ethical Committee of the Máxima Medical Centre in Veldhoven. All participants provided written informed consent prior to taking part in this study.

### Measures

#### Unplanned pregnancy

Participating women were asked about whether or not the current pregnancy was planned (yes/no), as part of the baseline questionnaire at 12 weeks into pregnancy. This way of assessment was chosen because of the specifics of the Dutch maternity system. The maternity system in the Netherlands is unique in comparison to other countries. Midwives work as independent healthcare professionals in small practices outside the hospital. Eighty percent of all pregnant women visit the community midwife for their first antenatal control at 8–12 weeks of gestation. However, in case a pregnancy is unplanned and unwanted, and a woman prefers an abortion, she visits a special abortion clinic for help at an earlier stage. As a result, most, if not all women, who eventually visit the midwife for the first antenatal control already have decided to accept the pregnancy, even if it was not planned. This perspective is supported in one of our previous studies conducted in the Netherlands (Pop et al. 2019), where this concept was defined as pregnancy was (1) planned, (2) unplanned but not unwanted, and (3) unplanned and unwanted. In this previous study, 7.9% of 1213 pregnancies were unplanned. Of these 95 unplanned pregnancies, only two (0.16% of the total group) were not wanted. Therefore, because this is such a small percentage, in the present study we decided to focus on the concept of “unplanned pregnancies” only.

#### Depressive symptoms

To assess depressive symptoms during pregnancy, the Dutch version of the Edinburgh Postnatal Depression Scale (EPDS) (Bergink et al. 2011; Cox et al. 1987; Pop et al. 1992) was administered at 12, 22, and 32 weeks of pregnancy and 1 week, 6 weeks, 4 months, 8 months, and 12 months postpartum. The EPDS is a ten-item scale measuring depressive symptoms over the past 7 days on a 4-point scale. Total scores range from 0 to 30, with higher scores indicating greater levels of depressive symptoms. The EPDS has been validated in pregnant women (Kozinszky and Dudas 2015), including a sample of Dutch pregnant women (Bergink et al. 2011). The EPDS is a valid and reliable instrument, both for use during pregnancy and postpartum (Bergink et al. 2011; Pop et al. 1992). A cutoff of ≥ 13 was used to categorize women with high levels of depressive symptoms indicating the presence of possible clinical depression, both during pregnancy and postpartum (Levis et al. 2020). The current study showed good internal consistency for all eight assessments, with Cronbach’s alphas ranging between 0.82 and 0.87.

#### Covariates

At 12 weeks of pregnancy, demographic characteristics were collected including age, level of education (high: Bachelor’s degree or higher), employment (yes/no), and having a partner (yes/no). Information regarding parity (primiparous/multiparous), previous miscarriage or abortion (yes/no), and a previous history of a depressive episode (yes/no) were also collected. Lifestyle habits were assessed by self-report at all trimesters of pregnancy, including information on body mass index (BMI), smoking (yes/no), and alcohol consumption during pregnancy.

### Statistical analyses

Differences in demographic and clinical characteristics between women with a planned pregnancy versus an unplanned pregnancy were examined using *t*-tests for continuous variables and chi-squared (*χ*^2^) tests for categorical variables. For the *χ*2 tests, the phi coefficient was calculated to establish the effect size (0.10 small, 0.30 medium, 0.50 large (Cohen 1988)). Furthermore, to examine group differences per time point, *t*-tests for all eight assessments were executed to test the differences in depressive symptom scores between women with a planned pregnancy versus an unplanned pregnancy. Next, mixed model analyses were used to investigate the association between pregnancy intention (planned versus unplanned) on the individual trajectories of depressive symptom levels over time, adjusting for covariates (i.e., age, BMI, level of education, employment, having a partner, parity, previous miscarriage or abortion, previous history of depressive episode, and smoking and alcohol consumption during pregnancy). A “null model” was created to fit the individual trajectories of depressive symptoms. We subsequently created a basic model by adding “time” as a continuous variable and “time” as a random slope. “Time” corresponds to the eight measurements of depressive symptoms throughout pregnancy and the postpartum period. With the random slope of time, a regression line is estimated for each participant. Based on maximum likelihood methods, the unstructured (UN) covariance matrix fitted the data best. Next, we created a predictor model by adding the variable unplanned pregnancy to the basic model. The next model included all covariates. In the final model, we tested the quadratic effect of time (time × time) and the interaction effects unplanned pregnancy × time and unplanned pregnancy × time × time. With the interaction effect time × time, it is tested whether there is a nonlinear, but quadratic, change in depressive symptom scores over time. The interaction effect pregnancy × time tests whether the change in depressive symptom scores over time is different for women with an unplanned pregnancy in comparison to women with a planned pregnancy. And finally, pregnancy × time × time is added to the model to test whether the possible quadratic change in depressive symptom scores over time is different for women with an unplanned pregnancy in comparison to women with a planned pregnancy. This resulted in a model including a random intercept, a random slope of time, the independent variable unplanned pregnancy plus all covariates, the quadratic effect of time (time × time), and the interaction effects unplanned pregnancy × time and unplanned pregnancy × time × time.

The Statistical Package for Social Sciences (SPSS version 24.0, IBM, Chicago, IL, USA) was used to conduct statistical analyses.

## Results

Table 1 shows the characteristics of both the participating women with an unplanned pregnancy (*N* = 111, 5.8%) and the comparison group of women with a planned pregnancy (*N* = 1817, 94.2%). For all significant demographic differences, the effect sizes ranged between small and medium.

**Table 1:** Characteristics of women who reported an unplanned pregnancy compared to those with planned pregnancy (*N* = 1928)

			Pregnancy intention					
	Total *N* = 1928		Unplanned *N* = 111 (5.8%)		Planned *N* = 1817 (94.2%)		*P-*value	
	*N* (%)	*Mean* ± *SD*	*N* (%)	*Mean* ± *SD*	*N* (%)	*Mean* ± *SD*	*χ*^2^	*t*-test
Age in years		30.47 ± 3.65		30.43 ± 5.10		30.48 ± 3.55		0.928
Level of education^1^							0.003**	
Low	686 (35.7)		54 (49.1)		632 (34.9)			
High	1233 (64.3)		56 (50.9)		1177 (65.1)			
Paid job	1803 (93.5)		94 (84.7)		1709 (94.1)		< 0.001***	
Partner	1906 (98.9)		100 (90.1)		1806 (99.4)		< 0.001***	
BMI pre-pregnancy (kg/m^2^)		23.81 ± 3.93		23.87 ± 4.07		23.81 ± 3.92		0.859
Alcohol use during pregnancy	77 (4.2)		7 (6.9)		70 (4.0)		0.166	
Smoking during pregnancy	109 (5.9)		21 (20.4)		88 (5.1)		< 0.001***	
Previous miscarriage/abortion	514 (26.7)		21 (18.9)		493 (27.1)		0.057	
Parity							0.122	
Primiparous	953 (49.6)		63 (56.8)		890 (49.2)			
Multiparous	967 (50.4)		48 (43.2)		919 (50.8)			
History of depression	292 (15.2)		34 (30.9)		258 (14.2)		< 0.001***	
EPDS 12 weeks of pregnancy		4.36 ± 4.18		6.40 ± 5.17		4.23 ± 4.08		< 0.001***
Above cutoff	108 (5.6)		16 (14.4)		92 (5.1)		< 0.001***	
EPDS 22 weeks of pregnancy		5.08 ± 4.18		6.95 ± 5.01		4.97 ± 4.10		< 0.001***
Above cutoff	115 (6.1)		17 (16.2)		98 (5.5)		< 0.001***	
EPDS 32 weeks of pregnancy		4.98 ± 4.17		6.96 ± 5.16		4.86 ± 4.08		< 0.001***
Above cutoff	104 (5.6)		15 (14.2)		89 (5.1)		< 0.001***	
EPDS 1 week postpartum		4.80 ± 4.75		5.99 ± 4.82		4.74 ± 4.74		0.023*
Above cutoff	122 (7.8)		8 (10.3)		114 (7.6)		0.399	
EPDS 6 weeks postpartum		4.94 ± 4.52		6.34 ± 4.67		4.86 ± 4.50		0.004**
Above cutoff	123 (7.4)		12 (14.5)		111 (7.1)		0.012*	
EPDS 4 months postpartum		4.76 ± 4.55		6.06 ± 4.57		4.69 ± 4.54		0.009**
Above cutoff	111 (6.9)		7 (9.0)		104 (6.8)		0.456	
EPDS 8 months postpartum		4.63 ± 4.63		6.56 ± 4.47		4.53 ± 4.62		< 0.001***
Above cutoff	112 (7.4)		10 (12.8)		102 (7.1)		0.060	
EPDS 12 months postpartum		4.47 ± 4.27		5.97 ± 4.30		4.39 ± 4.25		0.004**
Above cutoff	73 (5.6)		7 (11.1)		66 (5.3)		0.052	

BMI, body mass index; EPDS, Edinburgh Postnatal Depression Scale; SD, standard deviation

Percentages are valid percentages; for the EPDS, a cutoff of ≥ 13 was used; *p < 0.05; **p < 0.01; ***p < 0.001

^1^High, Bachelor or Master’s degrees; low, primary education; secondary pre-vocational education, secondary education or vocal education

### Depressive symptoms

**Fig. 2:**
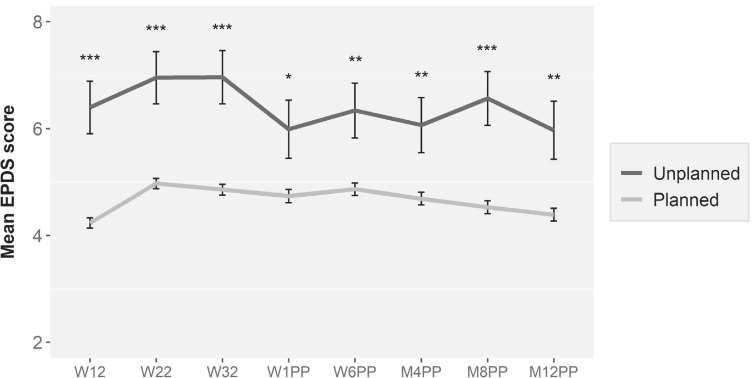
Depressive symptom mean scores at 12, 22, and 32 weeks of pregnancy and 1 week, 6 weeks, 4 months, 8 months, and 12 months postpartum in women with a planned and an unplanned pregnancy. EPDS, Edinburgh Postnatal Depression Scale; W, weeks; M, months; PP, postpartum. Error bars are standard error of the mean. *t*-tests: * *p* < 0.05, ** *p* < 0.01, *** *p* < 0.001

Mean EPDS scores, stratified for pregnancy intention, for all eight assessments during pregnancy and postpartum are shown in Table 1 (and visually in Fig. 2). Women who reported an unplanned pregnancy showed significantly higher levels of depressive symptoms compared to women with a planned pregnancy at all time-points (*t*-values varying from − 4.3 to − 2.3, *p* varying from < 0.001 to 0.023). Table 1 also shows the number of women scoring above the EPDS cutoff score for the presence of possible clinical depression. During the entire perinatal period (12 weeks of pregnancy up until 12 months postpartum), 460 (23.9%) women scored above the EPDS cutoff at least once. Of these 460 women, 41 (36.9%) belonged to the group of 111 women with an unplanned pregnancy and 419 (23.1%) to the group of 1817 women with a planned pregnancy, which is statistically significant (*χ*2 (1) = 11.1, *p* < 0.001).

### Linear mixed model analyses

In order to evaluate the course of depressive symptoms over time, discriminating between planned and unplanned pregnancy while adjusting for several covariates, we used linear mixed model analyses (Table 2). Women with an unplanned pregnancy had higher depressive symptom scores during the entire perinatal period compared to women with a planned pregnancy even after adjusting for various confounders (*p* < 0.001). The confounders lower age (*p* = 0.006), unemployment (*p* = 0.004), and history of depression (*p* < 0.001) were also significant predictors for perinatal depressive symptoms (Table 2). The beta coefficient of unplanned pregnancy (*β* =  − 2.24) can be explained as the percentage change in depressive symptom scores in women with an unplanned pregnancy, corresponding to 89.4% higher depressive symptom scores in women with an unplanned pregnancy compared to women with a planned pregnancy (formula: (expβ-1)*100). The model showed a significant effect of time (*p* = 0.049) and a significant quadratic effect of time (time × time, *p* = 0.022). Time showed a positive effect (*β* = 0.07), which means that the overall level of depressive symptoms increases over time. The negative interaction (*β* =  − 0.02) suggests that the steepness of this slope decreases over time, indicating that the increase of depressive symptom scores over time becomes less steep further into the postnatal period (a quadratic effect). However, the interaction terms unplanned pregnancy × time (*p* = 0.167) and unplanned pregnancy × time × time (*p* = 0.308) were not significant. This indicates that, even though there was a significant difference in depressive symptom levels between women with a planned and an unplanned pregnancy, the *course* of depressive symptom scores over time was the same for both groups.

**Table 2:** Linear mixed models with unplanned pregnancy as a predictor for depressive symptoms during the perinatal period

	Numerator df	Denominator df	*F*	*p*-value
Intercept	1	1992.671	58.114	< 0.001***
Unplanned pregnancy	1	5420.026	16.674	< 0.001***
Time	1	10,637.198	3.863	0.049*
Unplanned pregnancy × time	1	10,637.518	1.912	0.167
Time × time	1	10,177.936	5.258	0.022*
Unplanned pregnancy × time × time	1	10,178.167	1.038	0.308
Age	1	1811.030	7.635	0.006**
Level of education	1	1810.651	2.204	0.138
Paid job	1	1816.860	8.377	0.004**
Partner	1	1834.960	0.815	0.367
BMI pre-pregnancy	1	1805.323	2.577	0.109
Alcohol use during pregnancy	1	1823.576	0.643	0.423
Smoking during pregnancy	1	1835.531	2.860	0.091†
Previous miscarriage/abortion	1	1806.952	2.817	0.093†
Parity	1	1803.939	0.173	0.677
History of depression	1	1808.654	87.580	< 0.001***

†*p* < 0.10, *p < 0.05, **p < 0.01, ***p < 0.001

Because we observed a difference in the frequency of a history of depression between women with a planned pregnancy (14.2%) and women with an unplanned pregnancy (30.9%), we performed a sensitivity analysis repeating the mixed model analysis in a subgroup of women without a history of depression. This analysis showed similar results compared to that of the entire sample.

## Discussion

In the current study, we aimed to examine the association between an unplanned pregnancy and depressive symptoms in a large longitudinal cohort, stretching from early pregnancy until 12 months postpartum. Women who reported an unplanned pregnancy showed persistently higher levels of depressive symptoms in all trimesters of pregnancy and during the first 12 months postpartum. Lower age, unemployment, and a history of depression were independently related to higher levels of depressive symptoms reported during this period. We also showed that although women with an unplanned pregnancy showed persistently higher depressive symptom scores, the *course* of depressive symptom scores over time was similar to that of women with a planned pregnancy.

The association between unplanned pregnancy and depressive symptoms during the perinatal period has been reported in prior studies (e.g., Abajobir et al. 2016). However, almost all of these studies used a cross-sectional design or are limited by two time points. For example, Mercier et al. (2013) found that women with unintended pregnancies were more likely to have postpartum depression at both 3 and 12 months postpartum, and Faisal-Cury et al. (2017) found that unplanned pregnancy was associated with persistent maternal depression during the perinatal period. Our study adds a longitudinal aspect to the current literature, showing that higher levels of depressive symptoms persisted long after birth in women with unplanned pregnancy. In their longitudinal study, Christensen et al. (2011) did not find an association between unintended pregnancy and high depressive symptom levels during pregnancy; however, unintended pregnancy was associated with high postpartum depressive symptom levels. Nevertheless, compared to our sample, their study included a smaller sample (215 women), participants were included in a randomized controlled trial, and they used the Beck Depression Inventory-II (BDI-II) to screen for depressive symptoms. Furthermore, where Christensen et al. (2011) categorized pregnancy intention as intended, mistimed, and unwanted, the current study defined it as planned or unplanned pregnancy.

Another longitudinal study found that women with unwanted pregnancies, defined in terms of an unplanned *and* unwanted pregnancy and a negative first reaction to the pregnancy, reported slightly higher levels of perinatal depressive symptom scores; however, it diminished over the follow-up period (Najman et al. 1991). It may be difficult to compare the results of this study with the results of the current study. First, a different instrument, the Delusions-Symptoms-States Inventory (DSSI) was used to measure depressive symptoms. Secondly, the data of this study were collected between 1981 and 1984, almost 40 years ago. At that time, there might have been a different view on having an unplanned pregnancy and experiencing mental problems, which could have influenced the results of this study.

In the current study, 5.8% of the women reported an unplanned pregnancy, which corresponds to the national rates of the Netherlands in 2016, when 3% of the pregnancies were unplanned (similar for all levels of education) (RIVM 2017). These numbers are significantly lower than the number of unintended pregnancies found in studies from other countries, such as in the USA (45% unintended) (Finer and Zolna 2016) and the UK (16.2% unplanned, 29.0% ambivalent) (Wellings et al. 2013). Apart from the differences between the obstetric healthcare systems in different countries, another possible explanation for this difference could be the cultural differences in sexual education and contraception use. In many regions of the USA, the dominant sex education model focuses on abstinence-based ways to avoid pregnancy, whereas in the Netherlands, there are comprehensive and liberal school-based sex education programs. It has been shown that the sexual health–related outcomes (e.g., unwanted pregnancy rates and sexually transmissible infection statistics) are better in countries with sex-positive government policies (such as the Netherlands) in comparison to countries with a policy based on abstinence, such as the USA (Weaver et al. 2005). These cultural differences may therefore explain the low percentage of women reporting an unplanned pregnancy in the current study.

### Strengths and limitations

The major strengths of the current study are the large sample size (*N* = 1928) and the prospective longitudinal design assessing depressive symptoms at each trimester of pregnancy and five times postpartum enabling the use of mixed linear models. Other studies that assessed the relationship between an unplanned pregnancy and perinatal depressive symptoms mostly used a cross-sectional design or assessed depressive symptoms only twice during pregnancy or during the postpartum period. While these studies provide important information on the association between an unplanned pregnancy and depressive symptoms (Abajobir et al. 2016), the current study clearly demonstrates that an unplanned pregnancy seems to be a risk factor for heightened depressive symptom levels throughout the entire perinatal period until 12 months after giving birth.

This study also has limitations. The first limitation of this study is the assessment of an unplanned pregnancy. This study measured pregnancy intention by asking the question: “is the current pregnancy planned?” Women could indicate whether their current pregnancy was planned by selecting “yes” or “no.” In the Netherlands, most women who ask for an abortus provocatus because of an unwanted pregnancy visit a special abortion clinic before 8–10 weeks of gestation. Because in the current study community midwives included all participating women between 8 and 12 weeks, it is likely that most women with an unplanned and unwanted pregnancy have already made the decision for abortion. As a result, these women were not included in the study. Taken this background information into account, it is even more remarkable that an unplanned pregnancy — even when it is only unplanned but not unwanted — has a long-lasting negative impact on a woman’s perinatal mental health. Nonetheless, there is still much inconsistency in the literature about these terms and the exact definition of an unplanned pregnancy. The terms unplanned, unintended, mistimed, and unwanted pregnancy are often used interchangeably in the literature, yet there are important difference between these concepts (Goossens et al. 2016; Sedgh et al. 2014). Further studies are needed to address these differences in terminology. Barrett et al. (2004) developed the London Measure of Unplanned Pregnancy and Goossens et al. (2018) evaluated the psychometric properties of the Flemish version of this measurement. This measurement takes the complexity of pregnancy planning into account (example concepts: contraception use and pregnancy intention before becoming pregnant). It could be used in future studies, but it is also important to address the emotions that are associated with an unplanned pregnancy. A cross-sectional study by Barton et al. (2017) showed that women with an unplanned pregnancy, who had negative or ambivalent feelings toward their pregnancy, showed higher levels of psychological distress compared to women with an unplanned pregnancy that had happy feelings toward being pregnant.

Another limitation of the present study is that we assessed depressive symptoms by self-report instead of a diagnostic interview such as the SCID (First 2014). Similarly, history of depression was measured with a self-reported yes/no response. A more objective assessment of life-time history of depression would have given a better understanding of the concept, using a structural and standardized psychiatric diagnostic interview (e.g., CIDI, SCID). These instruments assessing the life-time history of depression is the golden standard allowing to discriminate between a major depressive episode and minor depression. Interview procedures are challenging in a longitudinal study like the current study with a large sample size (*N* = 1928). Furthermore, the DSM-5 highly advises that depressive symptoms are assessed regarding their intensity rather than using a dichotomous definition of depression only (Krueger et al. 2014).

Moreover, the participants of this study were more often highly educated, and most of them had a partner. Also fewer participants had an ethnic minority background compared to the general Dutch population (Statistics the Netherlands 2020, 2021). These factors may have had an attenuating effect on the level of depressive symptoms as related to unplanned pregnancy, and it is therefore difficult to generalize the present findings to other settings and countries. Additionally, social support was not considered in the present study, which is an important factor in the association of potentially negative experiences with perinatal depression (e.g., Biaggi et al. 2016). A final limitation of the study is that there is no data available on the use of antidepressants, psychotherapy, or psychological counseling for the participating women throughout the entire perinatal study period. Treatment for depression during the perinatal period may have reduced the level of depressive symptoms.

### Possible clinical implications

Our findings show strong support for the view that women with an unplanned pregnancy represent a group vulnerable to developing mental health problems. An important clinical implication could be that these women should be carefully followed, not only during pregnancy but also during the first postpartum year (and potentially beyond). Proactive screening of depressive symptoms followed by an intervention has repeatedly shown to be beneficial (O'Connor et al. 2016). Furthermore, perinatal depressive symptoms can lead to negative maternal mental health outcomes, such as increased level of parental stress (Misri et al. 2010), greater difficulties with social and partner relationships (Slomian et al. 2019), and suboptimal mother-to-infant bonding (Tichelman et al. 2019). The EPDS is a short and widely applicable instrument to screen for perinatal depression (O'Connor et al. 2016), which could easily be administered by midwives and obstetricians during pregnancy and by the general practitioners and infant healthcare workers during the first postpartum year.

## Data Availability

The datasets generated and analyzed during the current study are available from the corresponding author on reasonable request.

## References

[CR1] Abajobir AA, Maravilla JC, Alati R, Najman JM (2016). A systematic review and meta-analysis of the association between unintended pregnancy and perinatal depression. J Affect Disord.

[CR2] Bagiella E, Sloan RP, Heitjan DF (2000). Mixed-effects models in psychophysiology. Psychophysiol.

[CR3] Baron E, Bass J, Murray SM, Schneider M, Lund C (2017) A systematic review of growth curve mixture modelling literature investigating trajectories of perinatal depressive symptoms and associated risk factors. J Affect Disord 223:194-208.:10.1016/j.jad.2017.07.046 10.1016/j.jad.2017.07.046 PMC5592733 28763638

[CR4] Barrett G, Smith SC, Wellings K (2004). Conceptualisation, development, and evaluation of a measure of unplanned pregnancy. J Epidemiol Community Health.

[CR5] Barton K, Redshaw M, Quigley MA, Carson C (2017). Unplanned pregnancy and subsequent psychological distress in partnered women: a cross-sectional study of the role of relationship quality and wider social support. BMC Pregnancy Childbirth.

[CR6] Beck CT (2001). Predictors of postpartum depression: an update. Nurs Res.

[CR7] Bennett HA, Einarson A, Taddio A, Koren G, Einarson TR (2004). Prevalence of depression during pregnancy: systematic review. Obstet Gynecol.

[CR8] Van den Bergh BRH, van den Heuvel MI, Lahti M, Braeken M, de Rooij SR, Entringer S, Hoyer D, Roseboom T, Räikkönen K, King S, Schwab M (2017) Prenatal developmental origins of behavior and mental health: the influence of maternal stress in pregnancy. Neurosci Biobehav Rev.:10.1016/j.neubiorev.2017.07.003 10.1016/j.neubiorev.2017.07.003 28757456

[CR9] Bergink V, Kooistra L, Lambregtse-van den Berg MP, Wijnen H, Bunevicius R, van Baar A, Pop V (2011). Validation of the Edinburgh Depression Scale during pregnancy. J Psychosom Res.

[CR10] Biaggi A, Conroy S, Pawlby S, Pariante CM (2016). Identifying the women at risk of antenatal anxiety and depression: a systematic review. J Affect Disord.

[CR11] Boekhorst M, Beerthuizen A, Endendijk JJ, van Broekhoven KEM, van Baar A, Bergink V, Pop VJM (2019) Different trajectories of depressive symptoms during pregnancy. J Affect Disord 248:139–146.:10.1016/j.jad.2019.01.021 10.1016/j.jad.2019.01.021 30731281

[CR12] Christensen AL, Stuart EA, Perry DF, Le HN (2011). Unintended pregnancy and perinatal depression trajectories in low-income, high-risk Hispanic immigrants. Prev Sci.

[CR13] Cohen J (1988). Statistical power analysis for the behavioral sciences.

[CR14] Cox JL, Holden JM, Sagovsky R (1987) Detection of postnatal depression. Development of the 10-item Edinburgh Postnatal Depression Scale. Br J Psychiatry 150:782–786.:10.1192/bjp.150.6.782 10.1192/bjp.150.6.782 3651732

[CR15] Dibaba Y, Fantahun M, Hindin MJ (2013a) The association of unwanted pregnancy and social support with depressive symptoms in pregnancy: evidence from rural Southwestern Ethiopia. BMC Pregnancy and Childbirth 13 (1):135.:10.1186/1471-2393-13-135 10.1186/1471-2393-13-135 PMC3716614 23800160

[CR16] Dibaba Y, Fantahun M, Hindin MJ (2013b) The effects of pregnancy intention on the use of antenatal care services: systematic review and meta-analysis. Reprod Health 10:50.:10.1186/1742-4755-10-50 10.1186/1742-4755-10-50 PMC3848573 24034506

[CR17] Faisal-Cury A, Menezes PR, Quayle J, Matijasevich A (2017). Unplanned pregnancy and risk of maternal depression: secondary data analysis from a prospective pregnancy cohort. Psychol Health Med.

[CR18] Finer LB, Zolna MR (2016). Declines in unintended pregnancy in the United States, 2008–2011. N Engl J Med.

[CR19] First MB (2014) Structured clinical interview for the DSM (SCID). The encyclopedia of clin psychol. pp 1–6

[CR20] Gavin NI, Gaynes BN, Lohr KN, Meltzer-Brody S, Gartlehner G, Swinson T (2005) Perinatal depression: a systematic review of prevalence and incidence. Obstet Gynecol 106 (5 Pt 1):1071–1083.:10.1097/01.AOG.0000183597.31630.db 10.1097/01.AOG.0000183597.31630.db 16260528

[CR21] Goossens J, Van Den Branden Y, Van der Sluys L, Delbaere I, Van Hecke A, Verhaeghe S, Beeckman D (2016). The prevalence of unplanned pregnancy ending in birth, associated factors, and health outcomes. Hum Reprod.

[CR22] Goossens J, Verhaeghe S, Van Hecke A, Barrett G, Delbaere I, Beeckman D (2018) Psychometric properties of the Dutch version of the London Measure of Unplanned Pregnancy in women with pregnancies ending in birth. PLoS One 13 (4):e0194033.:10.1371/journal.pone.0194033 10.1371/journal.pone.0194033 PMC5905964 29668712

[CR23] Grote NK, Bridge JA, Gavin AR, Melville JL, Iyengar S, Katon WJ (2010). A meta-analysis of depression during pregnancy and the risk of preterm birth, low birth weight, and intrauterine growth restriction. Arch Gen Psychiatry.

[CR24] Hill B, Kothe EJ, Currie S, Danby M, Lang AY, Bailey C, Moran LJ, Teede H, North M, Bruce LJ, Skouteris H (2019) A systematic mapping review of the associations between pregnancy intentions and health-related lifestyle behaviours or psychological wellbeing. Prev Med Rep 14:100869.:10.1016/j.pmedr.2019.100869 10.1016/j.pmedr.2019.100869 PMC6465583 31011520

[CR25] Howard LM, Molyneaux E, Dennis CL, Rochat T, Stein A, Milgrom J (2014). Non-psychotic mental disorders in the perinatal period. Lancet.

[CR26] Kozinszky Z, Dudas RB (2015) Validation studies of the Edinburgh Postnatal Depression Scale for the antenatal period. J Affect Disord 176:95–105.:10.1016/j.jad.2015.01.044 10.1016/j.jad.2015.01.044 25704562

[CR27] Krueger RF, Hopwood CJ, Wright AG, Markon KE (2014) Challenges and strategies in helping the DSM become more dimensional and empirically based. Curr Psychiatry Rep 16 (12):515.:10.1007/s11920-014-0515-3 10.1007/s11920-014-0515-3 25308387

[CR28] Lancaster CA, Gold KJ, Flynn HA, Yoo H, Marcus SM, Davis MM (2010). Risk factors for depressive symptoms during pregnancy: a systematic review. Am J Obstet Gynecol.

[CR29] Levis B, Negeri Z, Sun Y, Benedetti A, Thombs BD (2020) Accuracy of the Edinburgh Postnatal Depression Scale (EPDS) for screening to detect major depression among pregnant and postpartum women: systematic review and meta-analysis of individual participant data. Bmj 371:m4022.:10.1136/bmj.m4022 10.1136/bmj.m4022 PMC7656313 33177069

[CR30] Liu Y, Kaaya S, Chai J, McCoy DC, Surkan PJ, Black MM, Sutter-Dallay AL, Verdoux H, Smith-Fawzi MC (2017). Maternal depressive symptoms and early childhood cognitive development: a meta-analysis. Psychol Med.

[CR31] McCrory C, McNally S (2013) The effect of pregnancy intention on maternal prenatal behaviours and parent and child health: results of an irish cohort study. Paediatr Perinat Epidemiol 27 (2):208-215.:10.1111/ppe.12027 10.1111/ppe.12027 23374066

[CR32] Mercier RJ, Garrett J, Thorp J, Siega-Riz AM (2013) Pregnancy intention and postpartum depression: secondary data analysis from a prospective cohort. Bjog 120 (9):1116-1122.:10.1111/1471-0528.12255 10.1111/1471-0528.12255 PMC3708972 23651010

[CR33] Misri S, Kendrick K, Oberlander TF, Norris S, Tomfohr L, Zhang H, Grunau RE (2010) Antenatal depression and anxiety affect postpartum parenting stress: a longitudinal, prospective study. Can J Psychiatry 55 (4):222-228.:10.1177/070674371005500405 10.1177/070674371005500405 20416145

[CR34] Najman JM, Morrison J, Williams G, Andersen M, Keeping JD (1991). The mental health of women 6 months after they give birth to an unwanted baby: a longitudinal study. Soc Sci Med.

[CR35] O'Connor E, Rossom RC, Henninger M, Groom HC, Burda BU (2016). Primary care screening for and treatment of depression in pregnant and postpartum women: evidence report and systematic review for the US Preventive Services Task Force. Jama.

[CR36] Pop VJ, Komproe IH, van Son MJ (1992). Characteristics of the Edinburgh Post Natal Depression Scale in the Netherlands. J Affect Disord.

[CR37] Pop V, van Son M, Wijnen H, Spek V, Denollet J, Bergink V (2019). Increase of depressive symptomatology during pregnancy over 25 years' time in four population based cohorts. J Affect Disord.

[CR38] Putnick DL, Sundaram R, Bell EM, Ghassabian A, Goldstein RB, Robinson SL, Vafai Y, Gilman SE, Yeung E (2020) Trajectories of maternal postpartum depressive symptoms. Pediatrics 146 (5).:10.1542/peds.2020-0857 10.1542/peds.2020-0857 PMC7772818 33109744

[CR39] RIVM (2017) Aanvullende Cijfers Seksuele Gezondheid.https://www.rivm.nl/leefstijlmonitor/aanvullende-cijfers-seksuele-gezondheid. Accessed 11 Jan 2022

[CR40] Robertson E, Grace S, Wallington T, Stewart DE (2004). Antenatal risk factors for postpartum depression: a synthesis of recent literature. Gen Hosp Psychiatry.

[CR41] Sedgh G, Singh S, Hussain R (2014). Intended and unintended pregnancies worldwide in 2012 and recent trends. Stud Fam Plann.

[CR42] Shay M, MacKinnon AL, Metcalfe A, Giesbrecht G, Campbell T, Nerenberg K, Tough S, Tomfohr-Madsen L (2020). Depressed mood and anxiety as risk factors for hypertensive disorders of pregnancy: a systematic review and meta-analysis. Psychol Med.

[CR43] Slomian J, Honvo G, Emonts P, Reginster JY, Bruyère O (2019) Consequences of maternal postpartum depression: a systematic review of maternal and infant outcomes. Womens Health (Lond) 15:1745506519844044.:10.1177/1745506519844044 10.1177/1745506519844044 PMC6492376 31035856

[CR44] Staneva A, Bogossian F, Pritchard M, Wittkowski A (2015). The effects of maternal depression, anxiety, and perceived stress during pregnancy on preterm birth: a systematic review. Women Birth.

[CR45] Statistics the Netherlands (2020) Levend geboren kinderen; huishoudenssamenstelling, regio [Children born alive; household compositions, region].https://opendata.cbs.nl/#/CBS/nl/dataset/82056NED/table?dl=15CD7. Accessed 10 Jan 2022

[CR46] Statistics the Netherlands (2021) Population; educational level; gender, age and migration background [Bevolking; onderwijsniveau; geslacht, leeftijd en migratieachtergrond].https://opendata.cbs.nl/statline/#/CBS/nl/dataset/82275NED/table?fromstatweb. Accessed 21 Jan 2021

[CR47] Tichelman E, Westerneng M, Witteveen AB, van Baar AL, van der Horst HE, de Jonge A, Berger MY, Schellevis FG, Burger H, Peters LL (2019) Correlates of prenatal and postnatal mother-to-infant bonding quality: a systematic review. PLoS One 14 (9):e0222998.:10.1371/journal.pone.0222998 10.1371/journal.pone.0222998 PMC6759162 31550274

[CR48] Truijens SE, Meems M, Kuppens SM, Broeren MA, Nabbe KC, Wijnen HA, Oei SG, van Son MJ, Pop VJ (2014) The HAPPY study (Holistic Approach to Pregnancy and the first Postpartum Year): design of a large prospective cohort study. BMC Pregnancy Childbirth 14:312.:10.1186/1471-2393-14-312 10.1186/1471-2393-14-312 PMC4162933 25201155

[CR49] Weaver H, Smith G, Kippax S (2005). School-based sex education policies and indicators of sexual health among young people: a comparison of the Netherlands, France, Australia and the United States. Sex Education.

[CR50] Wellings K, Jones KG, Mercer CH, Tanton C, Clifton S, Datta J, Copas AJ, Erens B, Gibson LJ, Macdowall W, Sonnenberg P, Phelps A, Johnson AM (2013). The prevalence of unplanned pregnancy and associated factors in Britain: findings from the third National Survey of Sexual Attitudes and Lifestyles (Natsal-3). Lancet.

[CR51] Woody CA, Ferrari AJ, Siskind DJ, Whiteford HA, Harris MG (2017). A systematic review and meta-regression of the prevalence and incidence of perinatal depression. J Affect Disord.

